# Bile pigments in emergency and critical care medicine

**DOI:** 10.1186/s40001-022-00863-0

**Published:** 2022-10-29

**Authors:** Mizuki Seya, Toshiyuki Aokage, Tsuyoshi Nojima, Atsunori Nakao, Hiromichi Naito

**Affiliations:** Department of Emergency, Critical Care, and Disaster Medicine, Okayama University Graduate School of Medicine, Dentistry, and Pharmaceutical Sciences, 2-5-1 Shikata-Cho, Kita-Ku, Okayama, 700-8558 Japan

**Keywords:** Bile pigments, Emergency and critical care medicine, Antioxidant therapy

## Abstract

Bile pigments, such as bilirubin and biliverdin, are end products of the heme degradation pathway in mammals and are widely known for their cytotoxic effects. However, recent studies have revealed that they exert cytoprotective effects through antioxidative, anti-inflammatory, and immunosuppressive properties. All these mechanisms are indispensable in the treatment of diseases in the field of emergency and critical care medicine, such as coronary ischemia, stroke, encephalomyelitis, acute lung injury/acute respiratory distress syndrome, mesenteric ischemia, and sepsis. While further research is required before the safe application of bile pigments in the clinical setting, their underlying mechanisms shed light on their utilization as therapeutic agents in the field of emergency and critical care medicine. This article aims to summarize the current understanding of bile pigments and re-evaluate their therapeutic potential in the diseases listed above.

## Background

Bile pigments, such as bilirubin (BR) and its oxidative derivative biliverdin (BV), are byproducts of the heme degradation pathway and are known largely as compounds assigned the job of excreting unwanted heme from the body. BR concentrations higher than the normal levels of 5 to 17 µM are known to exert cytotoxic effects [1]. However, recent studies have revealed that bile pigments within physiologically nontoxic concentrations demonstrate important potential anti-mutagenic, antioxidant, anti-inflammatory, and immunosuppressive properties [2]. In emergency and critical care medicine, where oxidative stress is closely tied to diseases, bile pigments have demonstrated therapeutic effects in both basic and clinical research. This review will give a synopsis of studies that infer the therapeutic effects of BV and BR in the field of emergency and critical care medicine. This article (i) examines the potential role of BV and BR as therapeutic agents, (ii) explores the roles of the already known cytoprotective effects of bile pigments in common diseases, and (iii) considers the potential clinical applications of BV in diseases.

## Reactive oxygen species (ROS) and their dual roles

There are two types of reactive oxygen species (ROS)—free radicals and nonradicals. Free radicals include the superoxide radical anion, nitric oxide, the carbonate radical anion, the hydroxyl radical, alkoxyl/alkyl peroxyl, and nitrogen dioxide. Hypochlorous acid, peroxynitrite/peroxynitrous acid, and hydrogen peroxide are the major nonradicals. Generation of ROS includes exogenous input and endogenous production from the mitochondrial electric transport chain, as well as the catalytic products of the enzymes NADPH oxidase, nitric oxide synthase, and myeloperoxidase. ROS can be changed from one type to another via a sequence of reactions. Excessive generation of ROS, primarily in the mitochondria, leads to damage to cells, tissues, and organs and is associated with pathogenesis in diabetes, cancer, cardiovascular, neurogenerative, reproductive system diseases, and the aging process [3].

ROS clearance depends on both enzymatic and nonenzymatic antioxidants. Enzymatic antioxidants include catalase, superoxide dismutase, the thioredoxin system, the glutathione peroxidase system, and peroxiredoxin. Nonenzymatic antioxidants include uric acid, glutathione, vitamin C, vitamin E, and bilirubin [4]. However, simply eliminating ROS is not as effective to restrain the development or progression of oxidative stress-induced damage. Homeostasis of cellular redox is meticulously balanced by the generation and elimination of ROS. ROS can not only cause oxidation of lipids, proteins, and DNA to damage cells but can also function as signaling molecules to modulate transcription factors and epigenetic pathways that determine cell survival and death. The dual nature of ROS explains this apparent paradox. Damaging ROS, such as hydroxyl radicals, are powerful oxidants that cause tissue damage, while beneficial species, such as hydrogen peroxide and superoxide, boost the mechanisms of endogenous antioxidant mechanisms via signal transduction pathways.

## Biliverdin/bilirubin as antioxidants

### The metabolic pathway of bilirubin and the heme degradation pathway

The metabolic pathway of bile pigments and the heme degradation pathway have led to the hypothesis that BR plays a main physiologic role as a cellular antioxidant [5]. In the heme degradation pathway, red blood cells are lysed by macrophages into the products heme and globin. Globin is then converted to amino acids, and heme is oxidized to carbon monoxide (CO), ferrous cation (Fe^2+^), and BV by heme oxygenase (HO). HO is a class of enzymes comprising constitutive and inducible isoforms, respectively, known as HO-1, 2, and 3. HO also exerts cytoprotective activities and signaling that regulate vasomotor tone, reduce inflammation and apoptosis, and bring immunomodulatory and antioxidant functions into play [6, 7]. While HO-2 and HO-3 are constantly present in the human body, HO-1 transcription is highly regulated by injurious stimuli, such as oxidative stress [8]. Thus, it is thought that the metabolites of HO-1, bile pigments, augment the anti-inflammatory and antioxidant effects of the HO-1 pathway.

**Fig. 1:**
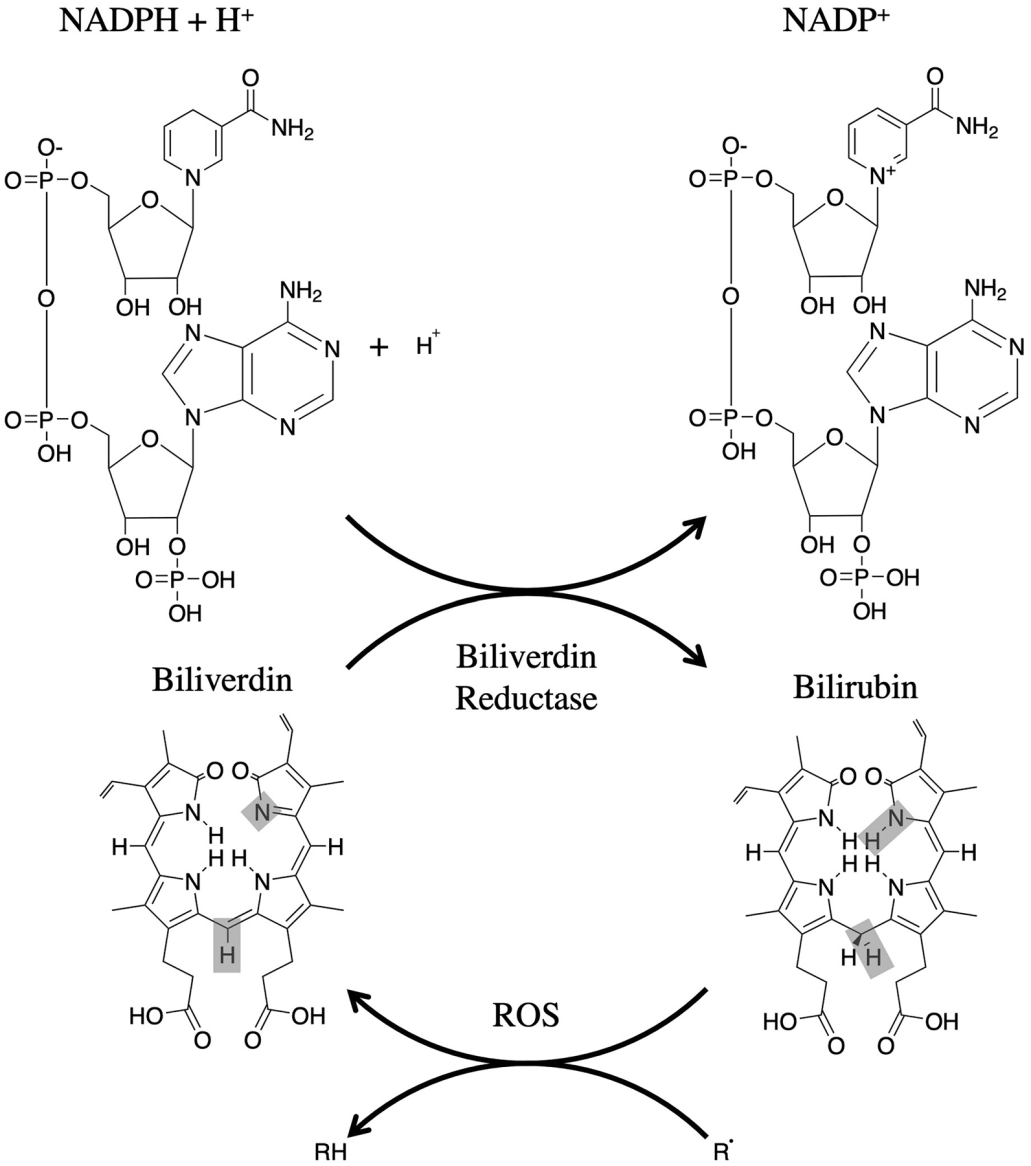
Bilirubin-biliverdin redox cycle. In the BR-BV redox cycle, BR reductase reduces BV by using NADPH and H + as an electron donor. This forms the products BR and NADP + . When BR is oxidized by ROS, BR reverts to become BV once again. This BV-BR redox cycle demonstrates the potential antioxidative properties of bile pigments in multiple disease models. (BR: bilirubin, BV: biliverdin, ROS: reactive oxygen species)

After heme degradation, BV is reduced to unconjugated BR by bilirubin reductase [2] (Fig. 1). At this stage, the unconjugated BR is water insoluble; thus, it binds to albumin to form an unconjugated BR albumin complex to be transferred to the liver through the bloodstream [9]. In the liver, uridine-diphosphoglucuronic glucuronosyltransferase conjugates the unconjugated BR to BR diglucuronide or BR monoglucuronide. This form of BR is water soluble, an essential characteristic for its elimination from the body. The conjugated BR is then secreted into the small intestine. It is then partially reabsorbed across the small intestine epithelium to be used in the liver again in the enterohepatic circulation. Unabsorbed conjugated BR continues through the digestive tract to be excreted. Once BR arrives at the distal ileum and colon, it is converted into urobilinogen by gut bacterial flora [9]. When oxidized, BR reverts to BV once again [2] (Fig. 1).

### Biliverdin/bilirubin cycle

Stoker et al. first demonstrated that conjugated BR at normal physiological concentrations plays an important role as an antioxidant [5, 10]. This is thought to take place through the BV/BR cycle, whereby BR reverts to become BV when oxidized and returns to BR when reduced by bilirubin reductase [2]. BR is a fascinating antioxidant because of its lipophilicity and its ubiquitous and cell-autonomous production from heme degradation that hampers lipid peroxidation [5]. The lipophilicity of unconjugated BR allows its passive diffusion through cellular membranes, including mitochondrial membranes [11]. BR can cross membranes by passive diffusion and equilibrate itself across the cytosol, mitochondria, and endoplasmic reticulum. When bilirubin scavenges H_2_O_2_, it is oxidized to form BV, which cannot easily cross membranes. Recently, Shum et al. showed that ATP-binding cassette sub-family B member 10 (ABCB10) is a mitochondrial BV exporter and demonstrated an essential transport process that intensifies intracellular BR redox actions [12].

## Dual effects of bilirubin/biliverdin

As shown in Table 1, bile pigments can be both beneficial and detrimental. Li et al. demonstrated that low-dose (2 mg/kg) BV led to depression-like behaviors, and high-dose (8 mg/kg) BV injection increased anxiety-like behaviors and impaired formation of memories in mice [13]. Thus, BR/BV have dual effects that may be caused by an imbalance in the redox system in tissues and cells, leading to oxidative stress. Although clear mechanisms were not determined, possible explanations are as follows. BV can have residual antioxidant, anti-inflammatory, and other neuroprotective effects at low doses, while BV in high doses is associated with nerve damage and inflammation, which can lead to anxious behaviors and depression. Additionally, intravenous injection of high doses of BV may result in negative HO-1 feedback regulation, which weakens HO-1-mediated antioxidant and anti-inflammatory effects. In addition, injection of too much exogenous BV may produce BR in the body, which plays a role in oxidative stress damage and central inflammation [13].

**Table 1:** Summary of studies that investigate the therapeutic potential and detrimental effects of bilirubin (BR) and biliverdin (BV)

Summary	Species	Disease	References
Detrimental effects			
Elevated BV levels are associated with increased mortality in patients with paracetamol-induced hepatic necrosis	Human	Paracetamol-induced hepatic necrosis	[61]
Neonatal exposure to high levels of BR causes severe motor symptoms and cerebral palsy	Human	Cerebral palsy	[62]
Unbound BR is strongly associated with auditory toxicity in neonates greater than or equal to 34 weeks of gestational age	Human	Auditory toxicity	[63]
Neonatal conditioning with concurrent hyperbilirubinemia and hypoxia-induced acidosis promoted long-term impairments in learning and memory complex sensorimotor functions. The toxicity is exacerbated through the activity of ASIC channels	Human, mouse	BR-induced neurotoxicity	[64]
Beneficial effects			
Intraperitoneal BR administration decreases infarct area in coronary I/R injury by preventing the oxidation of cardiolipin in the early cell death pathway	Rat	Cardiac I/R injury	[25]
BR nanoparticle administration ameliorate mRNA expression of inflammatory markers and monocyte infiltration during cardiac I/R injury	Mouse	Cardiac I/R injury	[28]
Intraperitoneal BV administration protected brain cells from oxidative injuries in MCA occlusion followed by reperfusion	Rat	Brain I/R injury	[32]
BR suppressed polyclonal and antigen-specific T cell responses in experimental autoimmune encephalomyelitis in mice	Mouse	Autoimmune Encephalomyelitis	[35]
BR nanoparticles delayed onset and progression of autoimmune encephalomyelitis and reduced the severity of the disease by acting on dendritic cells	Mouse	Autoimmune Encephalomyelitis	[36]
BV administration prevented gene expression of inflammatory mediators and gene fragmentation due to oxidative stress in acute lung injury/acute respiratory distress syndrome	Rat	Acute lung injury/acute respiratory distress syndrome	[38]
Hyperbilirubinemia ameliorated bleomycin-induced pulmonary fibrosis in rats	Rat	Bleomycin-induced pulmonary fibrosis	[41]
Bile pigment administration improved mesenteric I/R injury through its antioxidative properties	Rat	Mesenteric I/R injury	[43, 44]
BV administration achieves cytoprotective effects in mesenteric I/R injury through the suppression of NK-κB activation	Human	Mesenteric I/R injury	[46]
Intraperitoneal administration of BV improved host survival via the inhibition of inflammatory and oxidative markers, reduced neutrophil migration, and suppression of mucosal degradation	Rat	Intestinal transplantation	[49, 50]
BV maintained lung function post-transplantation and mitigated damage through the inhibition of inflammatory markers and MDA levels	Rat	Lung transplantation	[51]
BV administration ameliorated vascular endothelial injury caused by I/R and mechanical trauma	Rat	Vein graft and balloon injury	[52]
Hyperbilirubinemia prolonged graft survival and reduced acute rejection in heart transplantation	Rat	Cardiac transplantation	[53]
BV modulates inflammatory mediators and improves gastrointestinal function in polymicrobial sepsis	Rat	Sepsis	[54]
BV administration reduced endotoxemia-induced cellular damage in lungs and improved survival rates	Rat	Sepsis	[56]

## Therapeutic application in critical care/emergency medicine

### Bile pigments and cardiovascular diseases

Evidence now suggests that bile pigments act as inducers of the defensive mechanisms already existing in different types of ischemia–reperfusion (I/R) injury or cardiovascular disease [14–19]. Several studies show that serum BR levels are inversely proportional to atherosclerosis and cardiovascular disease risk in humans [6, 20, 21]. An example of this is a study showing that patients with Gilbert’s syndrome, a condition where a genetic variant induces hyperbilirubinemia [22], have a lower risk of coronary heart disease [17].

**Fig. 2:**
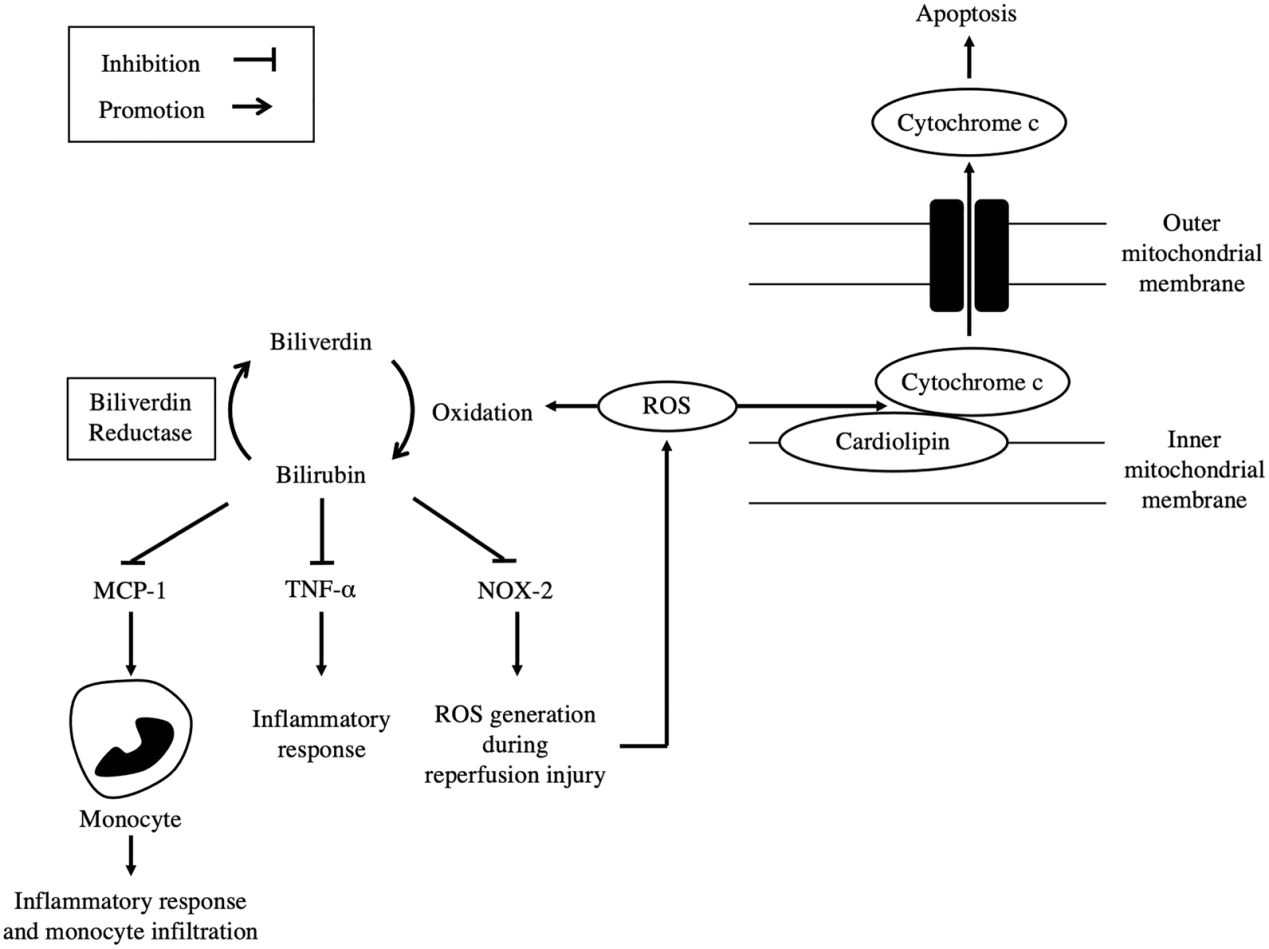
Bile pigments and cardiovascular diseases. CL is bound to cytc in the inner mitochondrial membrane and is released during the early stages of apoptosis in cardiomyocytes. Once oxidized by ROS, cytc leaves the mitochondria through the outer mitochondrial membrane and takes part in apoptosis. It is thought that the antioxidative properties of BR inhibit the release of cytc and in turn, prevent apoptosis in cardiomyocytes. Furthermore, BR administration is known to inhibit inflammation through downregulation of TNF-α, NOX-2, and MCP-1 during cardiac ischemia/reperfusion injury. (CL: cardiolipin, cytc: cytochrome c, ROS: reactive oxygen species, BR: bilirubin, TNF: tumor necrosis factor, NOX: nicotinamide adenine dinucleotide phosphate oxidase, MCP: monocyte chemoattractant protein)

Ben-Amotz et al. demonstrated that BR plays a role in preventing the oxidation of cardiolipin (CL) and the early cell death pathway, which in turn reduces infarct size in the cardiac muscle during ischemia. CL is bound to cytochrome *c* in the mitochondria and is released during the early stages of apoptosis by undergoing oxidation [23, 24]. BR’s antioxidant properties allow it to act as a cytoprotective molecule by inhibiting cytochrome *c* release from CL, leading to inhibition of the early stages of apoptosis in cardiomyocytes [25] (Fig. 2).

Elevated inflammatory cytokines are associated with cell apoptosis and tissue damage during coronary ischemia. Ai et al. found that BR nanoparticle (BRNP) administration ameliorated the mRNA expression of the inflammatory markers nicotinamide adenine dinucleotide phosphate oxidase-2 and tumor necrosis factor (TNF)-α during cardiac I/R. Monocyte infiltration during cardiac I/R is another factor in inflammatory damage [26] (Fig. 2). Studies by Tomczyk et al. revealed that HO-1 knockout led to the accumulation of higher numbers of macrophages in the cardiac muscle [27]. Monocyte chemoattractant protein (MCP)-1 was also revealed to be decreased in BRNP-administered cardiac I/R models [28]. The exact mechanism in which attenuation of inflammatory cytokines and cells is achieved during cardiac I/R is elusive. However, the results suggest that bile pigment administration into cardiac I/R models mitigates inflammation through actions such as the inhibition of monocyte infiltration.

Preconditioning therapies where bile pigments are administered before the onset of disease are impractical clinical treatments for human myocardial ischemia. However, in clinical situations where ischemia may be predicted, such as with grafts involving cardiac I/R injuries or interventional coronary procedures, BR pretreatment could potentially prevent cardiac damage.

### Bile pigments and stroke

Conventionally, bile pigments have been linked to neurologic dysfunction risk due to the preferential deposition of BR and the toxic effects of BR on cellular functions [29]. However, clinical studies reveal that high levels of serum BR have been linked to lower stroke risk and higher prevalence of unstable atherosclerotic regions [30, 31].

An in vivo study by Deguchi et al. used a rat model of cerebral infarction induced by transient occlusion of the middle cerebral artery (MCA) followed by reperfusion and reported that BV protected brain cells from oxidative injuries via its antioxidant efficacies. BV and vehicle were given intraperitoneally immediately after reperfusion. There was no significant difference in cerebral blood flow between the BV-administered and vehicle-administered groups. However, cerebral infarction size after two days of MCA occlusion had significantly decreased in BV-administered groups when evaluated using 2,3,5-triphenyltetrazolium chloride staining. Furthermore, in BV-administered specimens, superoxide generation in the cerebral cortex of the MCA area was significantly decreased. Staining with 8-hydroxy-2′-deoxyguanosine (8-OHdG) and 4-hydroxy-2-nonenal (4-HNE) was used to determine the oxidative impairment of neurons immunohistochemically. 8-OhdG and 4-HNE are DNA damage and lipid peroxidation markers, respectively. The quantity of stained cells in the cortex decreased significantly in BV-administered groups in comparison to vehicle-administrated groups. However, in both experiments, BV did not reduce superoxide generation and immunohistochemical oxidative impairment signals in the caudate as much as in the cortex, where there was a more severe form of ischemia. Thus, the nontoxic levels of BV used in this study are sufficient in reducing milder I/R injuries [32].

A recent study by Zou et al. suggests another mechanism of the protective effects of BV by the expressional network of microRNA (miRNA)-messenger RNA (mRNA) using a rat MCA occlusion model of cerebral I/R injury. The cerebral cortex was subjected to BV administration and ischemia, and mRNA and miRNA expressional profiles were studied using microarray technology. BV treatment upregulated the expression of miR-204 5p, miR-181b-5p, and miR-27a and induced the downregulation of their target genes to regulate the anti-inflammatory effect of BV in cerebral I/R. The miR-27a, miR-181b-5p, and miR-204 5p genes are, respectively, thought to be involved in cell proliferation, eosinophilic inflammation, and the anti-inflammatory pathway. Therefore, BV may affect crucial biological functions, like apoptosis, maintenance of adenosine triphosphate homeostasis, cell proliferation, and angiogenesis, by miRNAs regulating target genes [19].

### Bile pigments and encephalomyelitis

Encephalomyelitis is a life-threatening inflammation of the brain and spinal cord typically caused by infection with viruses, such as flaviviruses and alphaviruses. Recovery from encephalomyelitis is highly dependent on the body’s immune system [33]. BR has been demonstrated as a powerful immunomodulatory agent in research investigating the effects of bile pigments in encephalomyelitis.

**Fig. 3:**
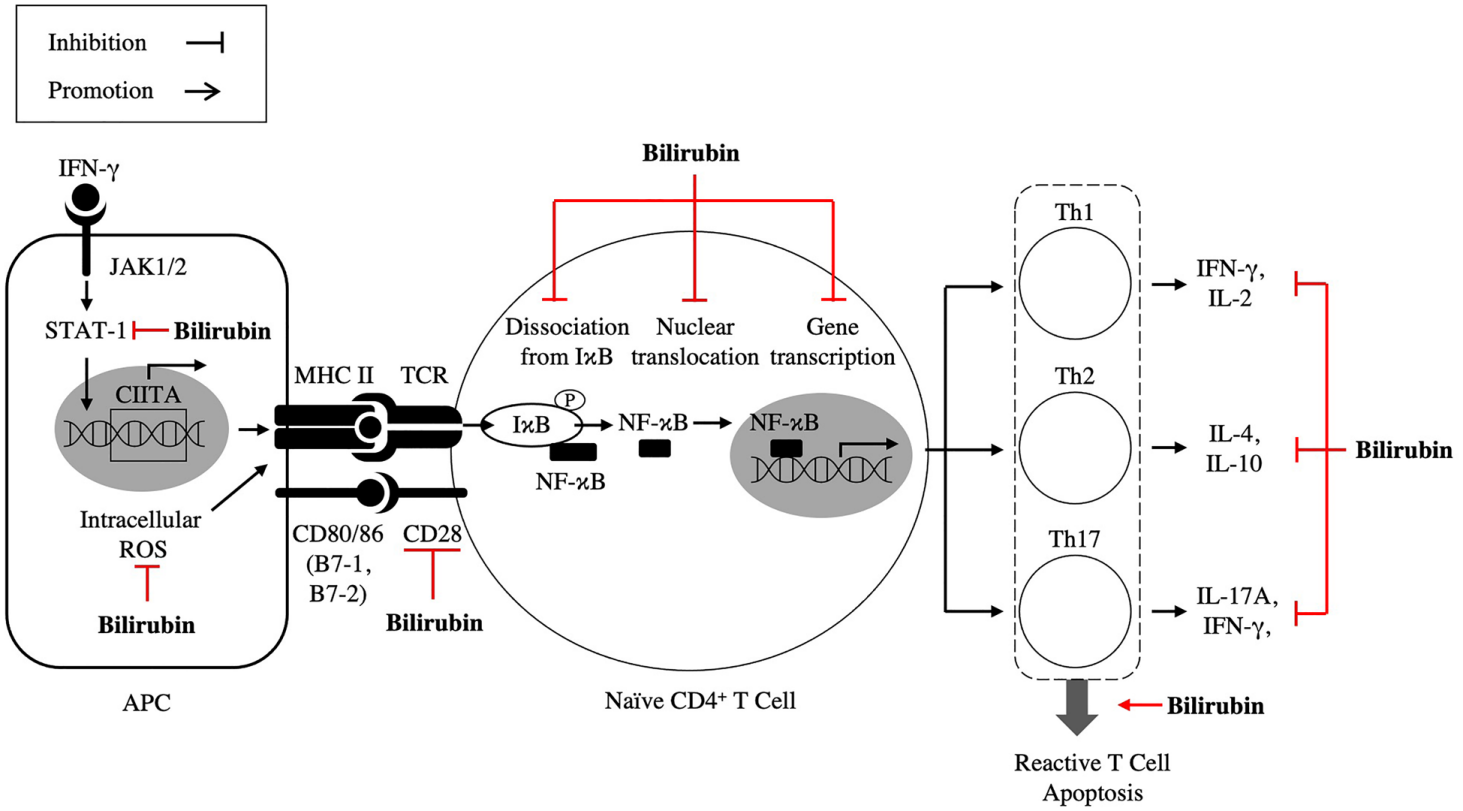
Bile pigments and encephalomyelitis. In encephalomyelitis, BR acts an important immunomodulator through multiple mechanisms, especially in T cell reactivity. BR inhibits STAT-1 and IκB phosphorylation, which are essential for CIITA and NF-κB activation in T cell activation. BR also achieves this via suppressing the upregulation of CD28, B7-1, and B7-2, and scavenging of ROS. Furthermore, BR treatment notably decreases the production of Th-1, Th-2, and Th-17 cytokines. Through these mechanisms, BR is thought to suppress T cell reactivity and act as a therapeutic agent in encephalomyelitis. (BR: bilirubin, NF-κB: nuclear factor kappa B, ROS: reactive oxygen species, Th: T helper cell)

The first reports of the effects of BR on immune system functions were published in the 1970s when Nejedlá et al. showed that hyperbilirubinemia suppressed antibody formation in newborns [34]. Liu et al. further showed in mice that BR effectively suppressed experimental autoimmune encephalomyelitis. In their paper, BR inhibited polyclonal and antigen-specific T cell responses, whereas other similar antioxidants did not. BR restrained CD4( +) T cell responses at several steps during an autoimmune reaction. For example, high levels of BR at 200 μM caused apoptosis in reactive CD4( +) T cells, and physiological BR concentrations led to anergy in reactive T cells. The mechanism of the phenomenon was determined to be achieved through a vast array of actions, such as inhibition of costimulatory activities, downregulation of inducible major histocompatibility complex (MHC) class II expression, and suppression of immune transcription factor activation. Furthermore, the production of both T helper cell (Th)-1 cytokines (IFN-γ, interleukin (IL)-2) and Th-2 cytokines (IL-4 and IL-10) were decreased by BR treatment of CD4 + cells (Fig. 3). In vivo research showed that BR treatment effectively curbed experimental autoimmune encephalomyelitis in mice, whereas depletion of endogenous BR seriously intensified this condition [35].

BR’s neuroprotective reactions in immune-mediated encephalomyelitis were achieved through ROS scavenging abilities. BRNP mitigates the progression of experimental autoimmune encephalomyelitis by negatively regulating the differentiation of naïve CD4 + T cells into Th17 cells by hindering the maturation of antigen-presenting cells (APCs) through scavenging of ROS overproduced in both macrophages and dendritic cells (DCs) upon uptake of Ag (Fig. 3). The therapeutic efficacy of BRNPs is due to (1) participating in pathogenic Th17 cell differentiation without systemic immunosuppression, (2) scavenging phagocytosis-induced ROS in macrophages and DCs, and (3) suppressing costimulatory molecule and MHC II expression on APCs ex vivo and in vivo [36, 37] (Fig. 3). Thus, these studies illustrate BR as a key immunomodulator that might protect mammals from neuroinflammatory and autoimmune diseases, giving evidence for its potential as a therapeutic agent in multiple disorders.

### Bile pigments and acute lung injury/acute respiratory distress syndrome

Acute lung injury/acute respiratory distress syndrome (ALI/ARDS) is another disease widely recognized as an important cause of poor outcomes in critically ill patients in the field of critical care and emergency medicine. A large part of the disease mechanism involves ROS production. Kosaka et al. explored the effects of administration of BV on ALI/ARDS induced by hemorrhagic shock and resuscitation (HSR) in rats. The lungs of BV-administered rats were histopathologically assessed, revealing reduced pulmonary edema and neutrophil migration. BV administration also significantly ameliorated gene expression of inflammatory mediators, such as TNF-α and inducible nitric oxide synthase. Immunosorbent assay and immunohistochemical analysis of 8-OHdG also indicated that BV administration reduced DNA fragmentation caused by oxidative stress. The findings above show that BV restrains HSR-induced ALI/ARDS through anti-inflammatory and antioxidant mechanisms [38].

ALI/ARDS can be split into two histopathological phases: the exudative phase and the fibroproliferative phase [39]. The fibroproliferative phase in ALI/ARDS is closely involved in the proliferation of fibroblasts and ameliorated pulmonary fibrosis has been known to improve gas exchange and mortality rates [40]. Wang et al.’s examination of hyperbilirubinemia in the development of bleomycin (BLM)-induced pulmonary fibrosis in rats allowed for the hypothesis that BR may be useful in improving long-term outcomes of ALI/ARDS [41]. BLM is a strong chemotherapeutic agent known to induce pulmonary fibrosis in both humans and experimental animals. The mechanism of BLM-induced fibrosis remains uncertain, but BLM-generated ROS is generally believed to cause direct injury to lung epithelial or endothelial cells [42]. All BR-administered BLM groups had significantly higher survival rates than the BLM group. Histopathological assessment showed that BV-administered groups had fewer infiltrations of inflammatory cells and fibrotic lesions when compared to groups treated with only BLM. Evaluation of bronchoalveolar lavage fluid (BALF) showed that hyperbilirubinemia inhibited BLM-induced increase in the numbers of lymphocytes and neutrophils. Markers of pulmonary fibrosis, hydroxyproline, and transforming growth factor-beta 1 were suppressed as well. Oxidative metabolite (BOM) urine content in the hyperbilirubinemia group was also examined to determine the oxidation of BR in disease models. BOM levels in the BR-administered groups and control groups did not change, whereas BOM levels in the BLM groups increased. These findings suggest that BR plays a protective role against fibrosis in the lung through its anti-inflammatory and antioxidative actions [41].

### Bile pigments and mesenteric ischemia

Mesenteric ischemia and reperfusion is a high mortality diagnosis in the field of emergency and critical care medicine that arises from etiologies that involve the mismatch of supply and demand in the mesenteric vessels. The pathophysiology involves the destruction of the villous layer of the intestinal mucosa, an increase in inflammatory mediators, like IL-1B and TNF-α, and decreased intestinal motility. Ceran et al. and Hammerman et al. showed that bile pigment administration in intestinal I/R models of rats mitigates histopathological grading and intestinal function. Furthermore, BV administration attenuates MDA and thiobarbituric acid-reducing substance levels, both byproducts of lipid peroxidation. This is indicative of the cytoprotective effects of bile pigments as antioxidants in settings of mesenteric I/R injury [43, 44].

A possible mechanism in which BV administration achieves cytoprotective effects in mesenteric I/R injury is through suppression of NF-κB activation, a transcriptional factor contributing to multiple functions in the body, such as immunity and inflammation [45]. Gibbs and Maines demonstrated that BV-treated cells had reduced NF-κB DNA binding in an electromobility shift assay. NF-κB upregulation via the inflammatory marker TNF-α is also suppressed by BV administration. This effect was further accentuated in a dose-dependent manner, whereby increased BV doses increased inhibitory activity [46]. Other products in the HO-1 pathway, such as HO-1 and CO, have been shown to alter NF-κB activation as well [47, 48]. All these studies suggest that BV’s anti-apoptotic and anti-inflammatory effect in mesenteric ischemia is achieved through NF-κB signaling and cross-interaction between the HO pathway byproducts.

### Bile pigments and transplantation

Transplantation improves longevity and quality of life in patients with end-organ diseases. Patients will often experience serious anatomical and physiological modifications where several precautions must be taken to maintain the patient’s prognosis. However, even with excruciating measures to prevent adverse effects post-transplantation, I/R injuries, vascular endothelial injury, and organ rejection remain an issue. Understanding possible mechanisms to prevent the above issues in transplantation at the beginning is key to improving transplant recipients' quality of life.

Nakao et al. reported that BV significantly improved the survival of rats receiving intestinal transplants when both graft donors and recipients were administered BV intraperitoneally. The ischemic phase during transplantation induces ROS production, which in turn damages the vascular epithelial cells and upregulates systemic inflammatory responses, often leading to multiorgan failure. However, after six hours after reperfusion, BV treatment inhibited the expression of inflammatory and oxidative markers, reduced neutrophil migration, and ameliorated mucosal degradation. Moreover, host survival over 14 days of observation was improved in BV-treated recipients and intestinal circular muscle and gut wall permeability were preserved to a greater extent. Further assessment of the long-term outcomes of the treatment is necessary, but its beneficial effects were maintained for over 24 h despite BV returning to control levels within 2 h [49]. Nojima et al*.* also reported similar results, demonstrating that intraluminal administration of BV preserved graft integrity following intestinal transplantation. They demonstrated that graft permeability was maintained in BV-treated intestinal grafts, and reduction of claudin-1 expression, a tight junction barrier protein, was mitigated [50]. Sugimoto et al. showed that BV administration ameliorated cold ischemia-induced lung damage in orthotopic left lung transplantation in rats. In this model, BV maintained gas exchange in the graft post-transplantation. They also showed that mRNA expression of inflammatory markers and MDA levels were reduced. Additionally, histopathological analysis of graft specimens exhibited decreased neutrophil migration and less anti-4-HNE antibody-positive cells in BV-treated graft specimens compared to the control group. The study surmises that the mechanism in which this is achieved occurs not only through ROS scavenging but also with the suppression of cascades of p-p38 mitogen-activated protein kinase (MAPK), p-ERK, and p-JNK, which are important pro-inflammatory cytokines [51].

Vascular endothelial injury caused by I/R and mechanical trauma during organ transplantation is another factor that attributes to recipient prognosis. Nakao et al. showed that BV treatment stops the formation of intimal hyperplasia caused by balloon angioplasty-induced vessel injury models or arterialized vein graft vasculopathy. Apoptotic rates of endothelial cells were reduced by 57% in BV-treated models and immunoblot analysis of MAPK activation showed that BV treatment suppressed JNK1/2 phosphorylation levels and *c*-Jun phosphorylation, which are known to be linked to cell death in I/R. In a wound migration assay, BV suppressed smooth muscle cell migration without loss of cellular viability, which is thought to be key in the development of intimal hyperplasia [52].

Despite the use of strong immunosuppressive agents, chronic graft rejection remains a problem in long-term patient and graft survival. Chronic graft rejection is defined as loss of allograft function several months after transplantation, and both antigen-dependent and -independent factors have been implicated to play a role in the disease mechanism. A study by S. Lee et al. explored how BR affected the long-term prognosis of heterotopic heart transplantation in either normobilirubinemic or hyperbilirubinemic rats under short-course tacrolimus immunosuppression. They demonstrated that acute rejection reduction and graft survival was prolonged in hyperbilirubinemic rats. Observation of histopathological specimen showed that BR reduced cell infiltration, fibrosis, and arteritis after 50 days post-transplantation; these were otherwise upregulated in normobilirubinamic rats. mRNA expression of inflammation (TNF-α, IL-6, and IL-1β) and Th1 type cytokines (IL-20 and IFN-γ) were also reduced at 30 days post-transplantation; signal transduction of p-extracellular signal-regulated kinase 1/2 activation was also attenuated. Lee et al. consider that this decrease in pro-inflammatory markers may have led to the downregulation of immunogenic influences, as demonstrated by the repression of anti-donor alloantibodies 60 days after an allogenic heart transplant. Modest inhibition of T cell proliferation was also noticeable in the presence of BR [53].

### Bile pigments and sepsis

Sepsis is an immune system overaction in response to an infection, and the disease mechanism involves overwhelming oxidative stress and pro-inflammatory responses along with diminished anti-inflammatory pathways. Overhaus et al. showed that BV exerts protection from polymicrobial sepsis by modulating inflammatory mediators [54]. The model for sepsis in this paper was created by cecal ligation and puncture (CLP) in rats grouped into samples that did or did not receive intraperitoneal BV injections. Their research revealed that treatment with BV improved gastrointestinal function and stopped sepsis-induced ileus, restoring the transit distribution pattern to nearly the control distribution pattern. They also observed upregulation of the anti-inflammatory mediators HO-1 and IL-10. IL-10 is a modulator of pro-inflammatory mediators, such as TNF-α and IL-6, and prevents nitric oxide generation by lipopolysaccharide (LPS)-activated monocytes/macrophages. Furthermore, BV treatment also decreased the induction of MCP-1 and IL-6 levels and reduced leukocyte infiltration into the small intestinal muscularis in CLP animals. MCP-1 is a mediator of macrophages during LPS-induced endotoxemia [55] and its suppression alongside the reduction of leukocyte infiltration suggests that BV is a key player in controlling inflammation during CLP-induced sepsis.

Sarady-Andrews et al. demonstrated that BV successfully reduced endotoxemia-induced cellular damage in the lungs and increased animals’ 24-h survival rate from 20 to 87% [56]. BALF assessment in pre-BV-treated specimens 24 h after the operation revealed suppression of LPS-induced neutrophil infiltration into the lung. This result was reproduced even when BV was administered after CLP-induced sepsis to simulate the clinical setting. Furthermore, they observed that the LPS-induced pro-inflammatory cytokine IL-6 was suppressed, and serum levels of IL-10 were elevated during BV treatment. In vitro analysis of mouse peritoneal macrophages and lung endothelial cells with LPS administration and BV treatment also revealed that IL-6 production was decreased in both cell types, which was associated with the decrease of NF-κB. Interestingly, this was the opposite effect of that observed in Overhaus et al.’s paper whereby BV further accentuated CLP-induced increase in NF-κB, [54], while Sarady-Andrews et al. showed that BV reduces NF-κB binding in the lung. However, our scientific understanding of NF-κB’s functional role is ever-evolving, and its function in different cell types in different models is yet to be revealed. Nonetheless, BV administration in sepsis and the NF-κB pathway is closely related to exertion of cytoprotective effects.

## Therapeutic application of bile pigments in clinical scenarios

Many investigators conducting research on bile pigments aim to benefit patients in clinical settings. Early preliminary clinical reports have demonstrated the effectiveness of Yutan and Goou, traditional Chinese drugs containing BV, for chronic liver disease [57]. Furthermore, a randomized study on healthy volunteers showed that the intravenous administration of hemin, a metabolic product of hemoglobin, safely induced HO-1 expression and activity [58]. Other research has used various strategies to utilize pharmacological HO-1 inducers, such as cobalt protoporphyrin and BRNPs [36, 37, 59, 60]. These studies overcome a fundamental hurdle in the clinical application of HO-1 and bile pigments. Although further toxicological and long-term studies are needed to determine the benefit and toxicity profile of BV before it can be used as a therapeutic agent in the clinic, evidence showing that bile pigments are potentially safe and effective treatments in the clinical setting is increasing.

## Conclusions

**Fig. 4:**
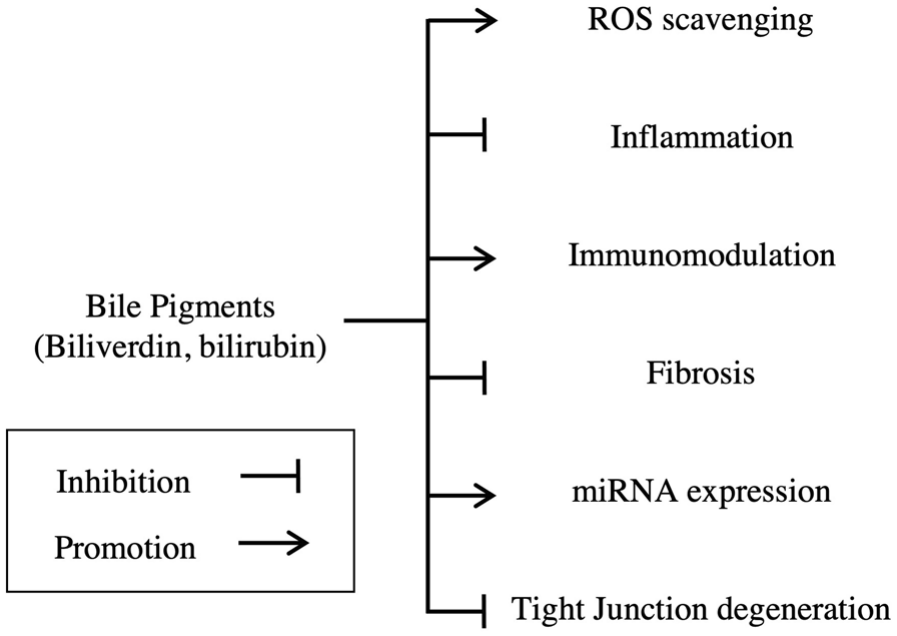
Summary of the mechanics of bile pigments in emergency and critical care medicine. The studies mentioned in this review demonstrate that the cytoprotective effects of bile pigments are achieved through its antioxidative, anti-inflammatory, and immunosuppressive properties, which are exhibited in multiple diseases of different etiologies. They are also known to inhibit fibrosis in chronic disease models and take part in miRNA expression and maintain cell integrity. Although many of these mechanisms are not clearly determined, cumulative and multifunctional therapeutic effects of bile pigments shed light on their utilization in the field of emergency and critical care medicine

Bile pigments are powerful agents that play a key clinical role in diseases prevalent in the field of emergency and critical care medicine. These studies demonstrate that the cytoprotective effects of bile pigments involve antioxidative, anti-inflammatory, and immunosuppressive properties, which are exhibited in multiple diseases of different etiologies (Fig. 4). While further research is required before the safe application of bile pigments in the clinical field, the cumulative and multifunctional therapeutic effects of bile pigments shed light on their utilization in the field of emergency and critical care medicine.

## Data Availability

Not applicable.

## Abbreviations

4-HNE: 4-Hydroxy-2-nonenal
8-OHdG: 8-Hydroxy-2′-deoxyguanosine
ALI/ARDS: Acute lung injury/acute respiratory distress syndrome
APCs: Antigen-presenting cells
BALF: Bronchoalveolar lavage fluid
BLM: Bleomycin
BOM: Urine content of oxidative metabolites
BR: Bilirubin
BRNP: Bilirubin nanoparticles
BV: Biliverdin
CO: Carbon monoxide
CL: Cardiolipin
CLP: Cecal ligation and puncture
DC: Dendritic cells
Fe^2+^: Ferrous cation
HO: Heme oxygenase
HSR: Hemorrhagic shock and resuscitation
IL: Interleukin
I/R: Ischemia–reperfusion
LPS: Lipopolysaccharide
MAPK: Mitogen-activated protein kinase
MCA: Middle cerebral artery
MCP: Monocyte chemoattractant protein
MHC: Major histocompatibility complex
miRNA: MicroRNA
mRNA: Messenger RNA
ROS: Reactive oxygen species
Th: T helper cell
TNF: Tumor necrosis factor

## References

[1] Asad SF, Singh S, Ahmad A, Khan NU, Hadi SM (2001). Prooxidant and antioxidant activities of bilirubin and its metabolic precursor biliverdin: a structure-activity study. Chem Biol Interact.

[2] McDonnell M, Mohiuddin S (2022). Biochemistry, biliverdin.

[3] Nickel A, Kohlhaas M, Maack C (2014). Mitochondrial reactive oxygen species production and elimination. J Mol Cell Cardiol.

[4] Zhang L, Wang X, Cueto R, Effi C, Zhang Y, Tan H (2019). Biochemical basis and metabolic interplay of redox regulation. Redox Biol.

[5] Stocker R, Yamamoto Y, McDonagh AF, Glazer AN, Ames BN (1987). Bilirubin is an antioxidant of possible physiological importance. Science.

[6] Ollinger R, Yamashita K, Bilban M, Erat A, Kogler P, Thomas M (2007). Bilirubin and biliverdin treatment of atherosclerotic diseases. Cell Cycle.

[7] Ryter SW, Tyrrell RM (2008). The heme synthesis and degradation pathways: role in oxidant sensitivity. Heme oxygenase has both pro- and antioxidant properties. Free Radic Biol Med..

[8] Wu B, Wu Y, Tang W (2019). Heme catabolic pathway in inflammation and immune disorders. Front Pharmacol.

[9] Kalakonda A, Jenkins B, John S (2022). Physiology, bilirubin.

[10] Tomaro ML, Batlle AM (2002). Bilirubin: its role in cytoprotection against oxidative stress. Int J Biochem Cell Biol.

[11] Zucker SD, Goessling W, Hoppin AG (1999). Unconjugated bilirubin exhibits spontaneous diffusion through model lipid bilayers and native hepatocyte membranes. J Biol Chem.

[12] Shum M, Shintre CA, Althoff T, Gutierrez V, Segawa M, Saxberg AD (2021). ABCB10 exports mitochondrial biliverdin, driving metabolic maladaptation in obesity. Sci Transl Med..

[13] Qaisiya M, Brischetto C, Jasprova J, Vitek L, Tiribelli C, Bellarosa C (2017). Bilirubin-induced ER stress contributes to the inflammatory response and apoptosis in neuronal cells. Arch Toxicol.

[14] Nakao A, Choi AM, Murase N (2006). Protective effect of carbon monoxide in transplantation. J Cell Mol Med.

[15] Nakao A, Neto JS, Kanno S, Stolz DB, Kimizuka K, Liu F (2005). Protection against ischemia/reperfusion injury in cardiac and renal transplantation with carbon monoxide, biliverdin and both. Am J Transplant.

[16] Nakao A, Kimizuka K, Stolz DB, Seda Neto J, Kaizu T, Choi AM (2003). Protective effect of carbon monoxide inhalation for cold-preserved small intestinal grafts. Surgery.

[17] Marconi VC, Duncan MS, So-Armah K, Re VL, Lim JK, Butt AA (2018). Bilirubin is inversely associated with cardiovascular disease among HIV-positive and HIV-negative individuals in VACS (Veterans Aging Cohort Study). J Am Heart Assoc.

[18] Clark JE, Foresti R, Sarathchandra P, Kaur H, Green CJ, Motterlini R (2000). Heme oxygenase-1-derived bilirubin ameliorates postischemic myocardial dysfunction. Am J Physiol Heart Circ Physiol.

[19] Zou ZY, Liu J, Chang C, Li JJ, Luo J, Jin Y (2019). Biliverdin administration regulates the microRNA-mRNA expressional network associated with neuroprotection in cerebral ischemia reperfusion injury in rats. Int J Mol Med.

[20] Morita T (2005). Heme oxygenase and atherosclerosis. Arterioscler Thromb Vasc Biol.

[21] Mehta NU, Reddy ST (2015). Role of hemoglobin/heme scavenger protein hemopexin in atherosclerosis and inflammatory diseases. Curr Opin Lipidol.

[22] Wagner KH, Shiels RG, Lang CA, Seyed Khoei N, Bulmer AC (2018). Diagnostic criteria and contributors to Gilbert's syndrome. Crit Rev Clin Lab Sci.

[23] Gonzalvez F, Gottlieb E (2007). Cardiolipin: setting the beat of apoptosis. Apoptosis.

[24] Orrenius S, Zhivotovsky B (2005). Cardiolipin oxidation sets cytochrome c free. Nat Chem Biol.

[25] Ben-Amotz R, Bonagura J, Velayutham M, Hamlin R, Burns P, Adin C (2014). Intraperitoneal bilirubin administration decreases infarct area in a rat coronary ischemia/reperfusion model. Front Physiol.

[26] Tomczyk M, Kraszewska I, Dulak J, Jazwa-Kusior A (2019). Modulation of the monocyte/macrophage system in heart failure by targeting heme oxygenase-1. Vascul Pharmacol.

[27] Tomczyk M, Kraszewska I, Szade K, Bukowska-Strakova K, Meloni M, Jozkowicz A (2017). Splenic Ly6C(hi) monocytes contribute to adverse late post-ischemic left ventricular remodeling in heme oxygenase-1 deficient mice. Basic Res Cardiol.

[28] Ai W, Bae S, Ke Q, Su S, Li R, Chen Y (2021). Bilirubin Nanoparticles Protect Against Cardiac Ischemia/Reperfusion Injury in Mice. J Am Heart Assoc.

[29] Wennberg RP (1991). Cellular basis of bilirubin toxicity. N Y State J Med.

[30] Matic LP, Jesus Iglesias M, Vesterlund M, Lengquist M, Hong MG, Saieed S (2018). Novel multiomics profiling of human carotid atherosclerotic plaques and plasma reveals biliverdin reductase B as a marker of intraplaque hemorrhage. JACC Basic Transl Sci.

[31] Choi Y, Lee SJ, Spiller W, Jung KJ, Lee JY, Kimm H (2020). Causal associations between serum bilirubin levels and decreased stroke risk: a two-sample mendelian randomization study. Arterioscler Thromb Vasc Biol.

[32] Deguchi K, Hayashi T, Nagotani S, Sehara Y, Zhang H, Tsuchiya A (2008). Reduction of cerebral infarction in rats by biliverdin associated with amelioration of oxidative stress. Brain Res.

[33] Griffin DE (2010). Recovery from viral encephalomyelitis: immune-mediated noncytolytic virus clearance from neurons. Immunol Res.

[34] Nejedla Z (1970). The development of immunological factors in infants with hyperbilirubinemia. Pediatrics.

[35] Liu Y, Li P, Lu J, Xiong W, Oger J, Tetzlaff W (2008). Bilirubin possesses powerful immunomodulatory activity and suppresses experimental autoimmune encephalomyelitis. J Immunol.

[36] Kim TW, Kim Y, Jung W, Kim DE, Keum H, Son Y (2021). Bilirubin nanomedicine ameliorates the progression of experimental autoimmune encephalomyelitis by modulating dendritic cells. J Control Release.

[37] Lee Y, Kim H, Kang S, Lee J, Park J, Jon S (2016). Bilirubin nanoparticles as a nanomedicine for anti-inflammation therapy. Angew Chem Int Ed Engl.

[38] Kosaka J, Morimatsu H, Takahashi T, Shimizu H, Kawanishi S, Omori E (2013). Effects of biliverdin administration on acute lung injury induced by hemorrhagic shock and resuscitation in rats. PLoS ONE.

[39] Luh SP, Chiang CH (2007). Acute lung injury/acute respiratory distress syndrome (ALI/ARDS): the mechanism, present strategies and future perspectives of therapies. J Zhejiang Univ Sci B.

[40] Meduri GU, Chinn AJ, Leeper KV, Wunderink RG, Tolley E, Winer-Muram HT (1994). Corticosteroid rescue treatment of progressive fibroproliferation in late ARDS. Patterns of response and predictors of outcome. Chest..

[41] Wang HD, Yamaya M, Okinaga S, Jia YX, Kamanaka M, Takahashi H (2002). Bilirubin ameliorates bleomycin-induced pulmonary fibrosis in rats. Am J Respir Crit Care Med.

[42] Hay J, Shahzeidi S, Laurent G (1991). Mechanisms of bleomycin-induced lung damage. Arch Toxicol.

[43] Hammerman C, Goldschmidt D, Caplan MS, Kaplan M, Bromiker R, Eidelman AI (2002). Protective effect of bilirubin in ischemia-reperfusion injury in the rat intestine. J Pediatr Gastroenterol Nutr.

[44] Ceran C, Sonmez K, Turkyllmaz Z, Demirogullarl B, Dursun A, Duzgun E (2001). Effect of bilirubin in ischemia/reperfusion injury on rat small intestine. J Pediatr Surg.

[45] Albensi BC (2019). What is nuclear factor kappa B (NF-kappaB) doing in and to the mitochondrion?. Front Cell Dev Biol.

[46] Gibbs PE, Maines MD (2007). Biliverdin inhibits activation of NF-kappaB: reversal of inhibition by human biliverdin reductase. Int J Cancer.

[47] Sarady JK, Otterbein SL, Liu F, Otterbein LE, Choi AM (2002). Carbon monoxide modulates endotoxin-induced production of granulocyte macrophage colony-stimulating factor in macrophages. Am J Respir Cell Mol Biol.

[48] Brouard S, Berberat PO, Tobiasch E, Seldon MP, Bach FH, Soares MP (2002). Heme oxygenase-1-derived carbon monoxide requires the activation of transcription factor NF-kappa B to protect endothelial cells from tumor necrosis factor-alpha-mediated apoptosis. J Biol Chem.

[49] Nakao A, Otterbein LE, Overhaus M, Sarady JK, Tsung A, Kimizuka K (2004). Biliverdin protects the functional integrity of a transplanted syngeneic small bowel. Gastroenterology.

[50] Nojima TNH, Obara T, Yamamoto H, Yumoto T, Igawa T, Aokage T, Seya M, Nakao A (2022). Luminal administration of biliverdin ameliorates ischemia/reperfusion injury following intestinal transplant in rats. Suregery..

[51] Sugimoto R, Tanaka Y, Noda K, Kawamura T, Toyoda Y, Billiar TR (2012). Preservation solution supplemented with biliverdin prevents lung cold ischaemia/reperfusion injury. Eur J Cardiothorac Surg.

[52] Nakao A, Murase N, Ho C, Toyokawa H, Billiar TR, Kanno S (2005). Biliverdin administration prevents the formation of intimal hyperplasia induced by vascular injury. Circulation.

[53] Lee S, Yamada T, Osako T, Stolz DB, Abe M, McCurry KR (2014). Recipient hyperbilirubinaemia protects cardiac graft in rat heterotopic heart transplantation. Eur J Cardiothorac Surg.

[54] Overhaus M, Moore BA, Barbato JE, Behrendt FF, Doering JG, Bauer AJ (2006). Biliverdin protects against polymicrobial sepsis by modulating inflammatory mediators. Am J Physiol Gastrointest Liver Physiol.

[55] Turler A, Schwarz NT, Turler E, Kalff JC, Bauer AJ (2002). MCP-1 causes leukocyte recruitment and subsequently endotoxemic ileus in rat. Am J Physiol Gastrointest Liver Physiol.

[56] Sarady-Andrews JK, Liu F, Gallo D, Nakao A, Overhaus M, Ollinger R (2005). Biliverdin administration protects against endotoxin-induced acute lung injury in rats. Am J Physiol Lung Cell Mol Physiol.

[57] Matsumoto N, Nakashima T, Kashima K (1995). Effectiveness of bovine gallstone (Goou) and bear gall powder (Yutan) on chronic liver diseases: a preliminary report. Tokai J Exp Clin Med.

[58] Bharucha AE, Kulkarni A, Choi KM, Camilleri M, Lempke M, Brunn GJ (2010). First-in-human study demonstrating pharmacological activation of heme oxygenase-1 in humans. Clin Pharmacol Ther.

[59] Liu X, Cui Y, Li M, Xu H, Zuo J, Fang F (2013). Cobalt protoporphyrin induces HO-1 expression mediated partially by FOXO1 and reduces mitochondria-derived reactive oxygen species production. PLoS ONE.

[60] Kim JY, Choi Y, Leem J, Song JE (2021). Heme oxygenase-1 induction by cobalt protoporphyrin ameliorates cholestatic liver disease in a xenobiotic-induced murine model. Int J Mol Sci..

[61] Wardle EN, Williams R (1981). Raised serum biliverdin in hepatic necrosis. Biochem Med.

[62] Rose J, Vassar R (2015). Movement disorders due to bilirubin toxicity. Semin Fetal Neonatal Med.

[63] Amin SB, Saluja S, Saili A, Laroia N, Orlando M, Wang H (2017). Auditory toxicity in late preterm and term neonates with severe jaundice. Dev Med Child Neurol.

[64] Lai K, Song XL, Shi HS, Qi X, Li CY, Fang J (2020). Bilirubin enhances the activity of ASIC channels to exacerbate neurotoxicity in neonatal hyperbilirubinemia in mice. Sci Transl Med.

